# Integrative Metabolome and Transcriptome Analyses Reveal the Effects of Plucking Flower on Polysaccharide Accumulation in the Rhizomes of *Polygonatum cyrtonema* Hua

**DOI:** 10.3390/molecules30030670

**Published:** 2025-02-03

**Authors:** Huidong Yang, Hua Li, Jiahui Huang, Xincheng Liu, Zhongdong Hu, Yi Liu

**Affiliations:** 1 Institute of Horticulture, Jiangxi Academy of Agricultural Sciences, Nanchang 330200, China; y17779300221@163.com (H.Y.); liuxch25@jxaas.cn (X.L.); 2 Jiangxi Key Laboratory of Horticultural Crops (Fruit, Vegetable & Tea) Breeding, Nanchang 330200, China; 3 Lushan Botanical Garden, Chinese Academy of Sciences, Jiujiang 332900, China; lihua083@163.com (H.L.); hjhui0015@163.com (J.H.); 4 Jiangxi Key Laboratory for Sustainable Utilization of Chinese Materia Medica Resources, Jiujiang 332900, China; 5 School of Life Sciences, Nanchang University, Nanchang 330031, China

**Keywords:** polysaccharides, rhizomes, *Polygonatum cyrtonema* Hua, metabolome and transcriptome, carbohydrate metabolic pathways, plucking flower

## Abstract

Polysaccharides are the major bioactive components of *Polygonatum cyrtonema* Hua, and their biosynthesis and accumulation are influenced by many agronomic practices. In this study, we applied integrative metabolome and transcriptome analyses to investigate the accumulation of bioactive components in one-year-old (1Y) and six-year-old (6Y) rhizomes of *P. cyrtonema* Hua treated with a plucking flower. The compound content analysis suggested that six-year-old treated rhizomes (T6) accumulated the highest polysaccharide content compared to that of one-year-old treated rhizomes (T1), one-year-old untreated rhizomes (C1), and six-year-old untreated rhizomes (C6). Metabolomics analysis showed that 4-*O*-galactopyranosylxylose, 6-*O*-α-l-arabinopyranosyl-d-glucopyranose, d-arabinose and dl-xylose significantly accumulated in T6 rhizomes. Carbohydrate metabolic pathways, including “glycolysis/gluconeogenesis”, “pentose and glucoronate interconversions” and “amino sugar and nucleotide sugar metabolism” were highly correlated with polysaccharide biosynthesis and accumulation. The transcriptome data indicated that *UPG2*, *GPI,* and *GALE* were positively upregulated in T6_vs_C6. In parallel, *RHM* and *PEI* were down-regulated in T6_vs_C6. Taken together, this study not only indicates that the candidate metabolites/metabolic pathways and genes affected by plucking flowers may influence the accumulation of polysaccharides in the rhizomes but also provides an easy and feasible agronomic practice to facilitate the accumulation of polysaccharides in the rhizomes of *P. cyrtonema* Hua.

## 1. Introduction

Polygonati rhizoma (PR), also known as *Huangjing* in China, is a well-known ingredient in traditional Chinese medicine and healthy food diets [1]. It is the rhizome of *P. sibiricum* Red, *P. cyrtonema* Hua, and *P. kingianum* Coll. et Hemsl, according to the record of the Chinese Pharmacopoeia (2020 edition). Various bioactive ingredients, including polysaccharides, triterpene saponins, steroidal saponins, flavonoids, and alkaloids, are responsible for the beneficial effects of PR [2]. In particular, polysaccharides have been identified as a quality marker of PR, and the polysaccharide content of qualified PR is more than 4% (*w*/*w*), according to the Chinese Pharmacopoeia (2020 edition). Among these three species, *P. cyrtonema* Hua showed the highest quality and efficacy owing to its relatively high polysaccharide content [3]. Polysaccharides in the genus *Polygonatum* are composed of a wide range of structural forms, including glucofructans, fructans, and pectins [4]. Polysaccharides have been demonstrated to possess a wide range of bioactivities, including anti-aging, antioxidant, immunomodulatory, anti-fatigue, anti-inflammatory, anti-diabetic, anti-osteoporotic, neuroprotective, anti-tumor, and kidney-protective effects [1,5]. Therefore, PR has attracted increasing attention from the food and health industries [6]. 

In recent years, the cultivation of *P. cyrtonema* Hua has shown considerable success, with a significant expansion of large-scale plantings in China. During the management process, many agronomic practices were employed. Among these practices, plucking flowers is a very simple agronomic practice that can significantly enhance the yield of forest fruits, vegetables, and traditional Chinese medicinal herbs. For example, flower thinning results in the growth of larger individual fruits on apple trees [7]. In *Salvia miltiorrhiza*, moderate plucking of flowers can effectively facilitate dry matter accumulation in the roots and increase yield [8]. For medicinal herbs, plucking flowers increased lutein content in three cultivars of *Abelmoschus esculentus* [9]. In addition, plucking flowers in *P. cyrtonema* Hua can reduce nutrient demands for reproductive growth and promote the transport and allocation of nutrients and carbon assimilation of rhizomes [10]. These studies provide a scientific basis for increasing the quality and yield by removing the inflorescences of traditional Chinese medicinal herbs, which significantly inhibits their reproductive growth and promotes the vegetative growth of plants. Recently, many genes involved in polysaccharide biosynthesis in *P. cyrtonema* Hua have been identified [11,12]. Polysaccharides are biosynthesized from monosaccharides by continuous enzymatic reactions, and the total polysaccharide content was positively correlated with the expression patterns of GDP-l-fucose synthase (*TSTA3*), UDP-apiose/xylose synthase (*AXS*), and UDP-glucose 6-dehydrogenase (*UGDH*) in *P. cyrtonema* Hua [12,13]. However, it remains unclear whether plucking flowers affects the accumulation of polysaccharides and how this practice affects genes and metabolites in the rhizomes of *P. cyrtonema* Hua.

In the present study, four types of samples were examined, including one-year-old (1Y) and six-year-old (6Y) rhizomes of the control group, which were designated C1 and C6, and one-year-old (1Y) and six-year-old rhizomes (6Y) of the plucking flower treatment group, which were designated T1 and T6, respectively. It was found that plucking flowers significantly increased the polysaccharide content in T6 compared to C1, T1, and C6. In order to illustrate the mechanism by which plucking flowers affect the accumulation of polysaccharides in rhizomes of *P. cyrtonema* Hua, UPLC–MS/MS-based widely targeted metabolomics and RNA-seq analyses were performed. Integrative metabolome and transcriptome analyses suggested that the up-regulation of UTP-glucose-1-phosphate uridylyltransferase (*UPG2*), UDP-glucose 4-epimerase (*GALE*) and Glucose-6-phosphate isomerase (*GPI*) in T6_vs_C6, and down-regulation of UDP-glucose 4,6-dehydratase (*RHM*) in T6_vs_C6 by plucking flowers may facilitate the biosynthesis and accumulation of metabolites d-arabinose and dl-xylose, thereby leading to the high accumulation of polysaccharides in T6. Thus, this study not only demonstrated that plucking flowers is an easy, feasible agronomic practice to increase the accumulation of polysaccharides in PR, but also provided an insight into the mechanism of how metabolites and genes affect the accumulation of polysaccharides in PR treated with plucking flowers. 

## 2. Results

### 2.1. Total Polysaccharide Content in the Rhizomes of P. cyrtonema Hua

**Figure 1:**
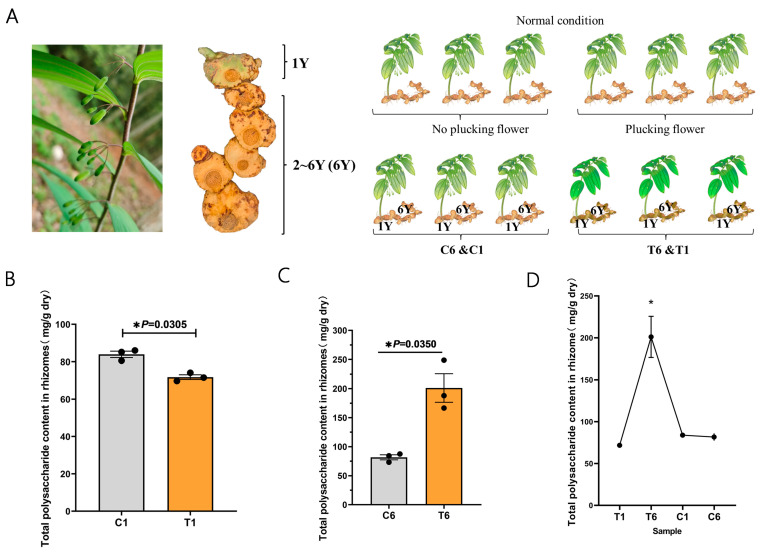
Schematic diagram of the experimental design and total polysaccharide content in the 1Y and 6Y rhizomes of *P. cyrtonema* Hua. (**A**) Schematic diagram of the experimental design. (**B**) Total polysaccharide content in 1Y rhizomes with and without plucking flower treatment. (**C**) Total polysaccharide content in 6Y rhizomes with and without plucking flower treatment. (**D**) Trends of total polysaccharide content from T1, T6, C1, and C6. “1Y” represents one-year-old rhizomes of *P. cyrtonema* Hua, “6Y” represents two- to six-year-old rhizomes of *P. cyrtonema* Hua, “C” for control, and “T” for plucking flower treatment. Error bars represent the standard deviation (n = 3). The data were processed using a t-test, with the following significance probabilities: * *p* < 0.05.

Total polysaccharides in the 1Y and 6Y rhizomes were separately extracted from the rhizomes of *P. cyrtonema Hua* with and without plucking flower treatment (Figure 1A). The total polysaccharide content showed a slight but significant reduction in the T1 rhizomes when compared with that of C1 rhizomes, with the lowest value of 71.78 ± 1.28 mg/g (Figure 1B). However, the total polysaccharide content significantly increased in T6 rhizomes, which showed the highest value (201.1 ± 24.61 mg/g) (Figure 1C). Overall, the total polysaccharide content of T1, C1, and C6 rhizomes was comparable, and the total polysaccharide content of T6 rhizomes showed a significant increase (Figure 1D). These results suggested that plucking flowers can significantly increase the total polysaccharide content of the 6Y rhizome of *P. cyrtonema* Hua.

### 2.2. Widely Targeted Metabolomics Analysis

**Figure 2:**
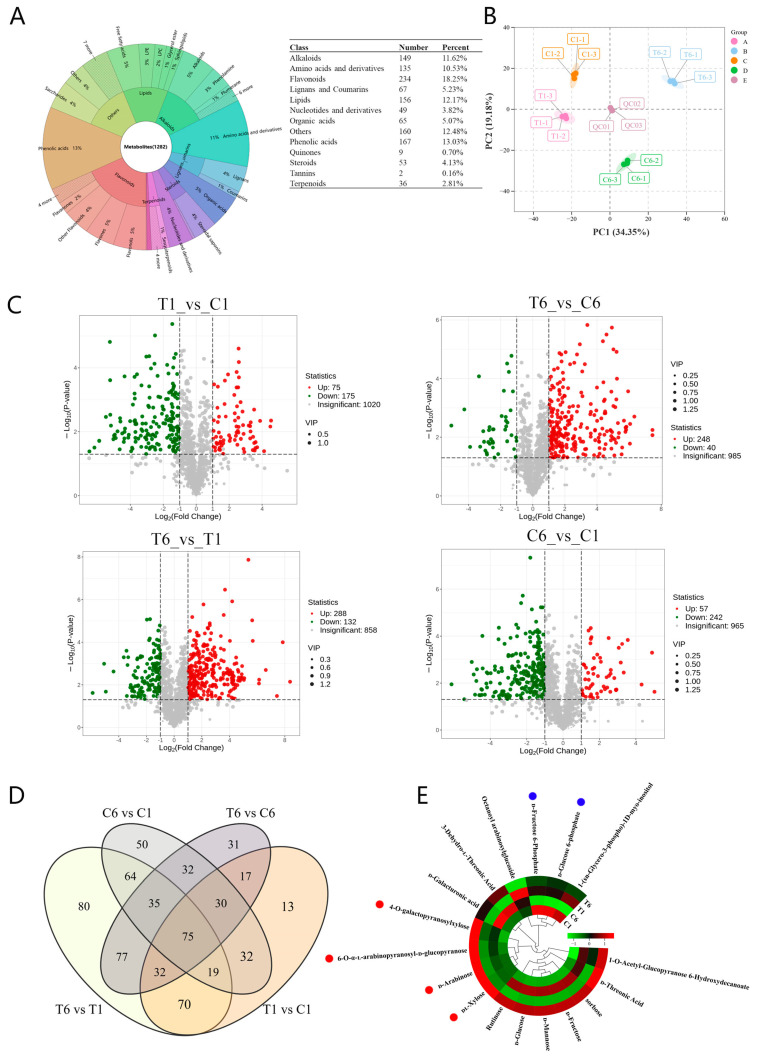
Widely targeted metabolomic analysis of rhizomes from C1, T1, C6, and T6 of *P. cyrtonema* Hua. (**A**) Classification of all identified metabolites. (**B**) Principal component analysis (PCA) score plots of metabolite profiles from four different groups. (**C**) Volcano plots of upregulated and downregulated differential metabolites from the four different groups. (**D**) Venn diagram of all significant metabolites among the four compared groups. (**E**) Heatmap analysis of 17 saccharide metabolites. In (**B**), groups A to E represent the samples of T1, T6, C1, C6, and QC, respectively; the QC data points at the plot center are the “quality/quantity control”; PCA is mainly used to judge the quality of biological repeatability within the group as a whole and the size of the difference between the groups. In (**C**), the metabolites of VIP (variable important in projection) > 1 were significantly different. Red dots in (**E**) represent the metabolites that significantly increase in T6 rhizomes; blue dots in (**E**) represent the metabolites that decrease in T1 rhizomes.

To further analyze the compound differences in total polysaccharide contents in T1_vs_C1 and T6_vs_C6, widely targeted metabolomics analysis was conducted using UPLC−MS/MS. A total of 1282 metabolites were obtained from all samples, which belonged to 13 classes: alkaloids (149), amino acids and derivatives (135), flavonoids (234), lignans and coumarins (67), lipids (156), nucleotides and derivatives (49), organic acids (65), phenolic acids (167), quinones (9), steroids (53), tannins (2), terpenoids (36), and other classes of metabolites (160) (Figure 2A). According to principal component analysis (PCA), the samples were separated into four groups, in which principal component 1 (PC1) accounted for 34.35% and principal component 2 (PC2) for 19.18%, to explain the overall variance and separation, respectively (Figure 2B). Volcano plots showed all differential expression metabolites (DEMs) for T1_vs_C1, T6_vs_C6, T6_vs_T1, and C6_vs_C1, which were 250 DEMs (175 downregulated and 75 upregulated), 288 DEMs (40 downregulated and 248 upregulated), 420 DEMs (132 downregulated and 288 upregulated), and 299 DEMs (242 downregulated and 57 upregulated), respectively (Figure 2C). It is noteworthy that more upregulated metabolites and fewer downregulated metabolites were discovered in T6_vs_C6 than in T1_vs_C1. A total of 657 DEMs were found in all four groups (Table S1), of which 75 DEMs were common among the four groups (Figure 2D). A total of 174 DEMs were present in only one comparison group, and the T6_vs_T1 group had the largest number of unique metabolites (80), followed by C6_vs_C1 (50), T6_vs_C6 (31), and T1_vs_C1 (13) (Figure 2D). Of the 657 DEMs, 17 DEMs were classified as saccharides, which can affect the biosynthesis and accumulation of polysaccharides. Heatmap analysis of these 17 DEMs showed that the contents of 4-*O*-galactopyranosylxylose, 6-*O*-α-l-arabinopyranosyl-d-glucopyranose, d-arabinose and dl-xylose significantly increased in T6 rhizomes, while the contents of d-fructose 6-phosphate and d-glucose 6-phosphate slightly decreased in T1 rhizomes (Figure 2E, Table S1).

**Figure 3:**
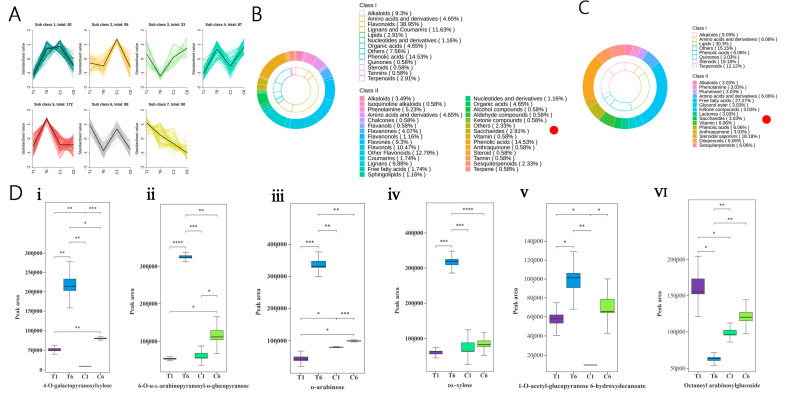
DEMs clustering analysis and relative levels of six saccharides from C1, T1, C6, and T6 in *P. cyrtonema* Hua. (**A**) Variation tendencies among the seven subclasses of all metabolites with significantly different contents in at least two samples. (**B**,**C**) Metabolites were classified into subclass 5 and subclass 3. (**D**) (i)~(vi) represent the peak area of six saccharide compounds in rhizomes of *P. cyrtonema* Hua from different treatments. Red dots in (**B**,**C**) represent saccharide compounds, and ClassⅠand classⅡrepresent primary classification of substances and secondary classification of substances; error bars in (**D**) represent standard deviation (n = 3); the data were processed by t-test, with the following significance probabilities: *, *p* < 0.05; **, *p* < 0.01; and ***, *p* < 0.001; ****, *p* < 0.0001.

According to the variation tendency of DEMs among the four compared groups, clustering analysis was carried out, and the DEMs were grouped into seven subclasses (Figure 3A). The changing pattern of metabolites in subclass 5 showed similar trends, and subclass 3 showed reverse trends to that of the total polysaccharide content in the rhizomes with and without plucking flower treatments (Figure 1D). Notably, the 1-*O*-acetyl-glucopyranose 6-hydroxydecanoate, 4-*O*-galactopyranosylxylose, 6-*O*-α-l-arabinopyranosyl-d glucopyranose, d-arabinose, and dl-xylose associated with saccharides were obtained from subclass 5 (Figure 3B), which were consistent with the result of heat map analysis (Figure 2E). Except for 1-*O*-acetyl-glucopyranose 6-hydroxydecanoate (Figure 3D(v)), the contents of the other four saccharides (4-*O*-galactopyranosylxylose, 6-*O*-alpha-l-arabinopyranosyl-d-glucopyranose, d-arabinose and dl-xylose) in T6 were statistically higher than those in C6 rhizomes (Figure 3D(i–iv)). In subclass 3, octanoyl arabinosylglucoside was obtained (Figure 3C). The production of octanoyl arabinosylglucoside in T6 rhizomes was significantly lower than that in C6 rhizomes (Figure 3D(vi)). This result suggested that octanoyl arabinosylglucoside might be a precursor of polysaccharides, which can be converted to polysaccharides under plucking flower treatment. Taken together, the metabolome analysis data demonstrated that these five saccharides (4-*O*-galactopyranosylxylose, 6-*O*-α-l-arabinopyranosyl-d-glucopyranose, d-arabinose, dl-xylose, and octanoyl arabinosylglucoside) may participate in the polysaccharide metabolism and contribute to the accumulation of polysaccharide treated with plucking flower in *P. cyrtonema* Hua rhizomes.

### 2.3. Transcriptome Analysis

#### 2.3.1. De Novo Transcriptome Assembly and Functional Annotation

**Figure 4:**
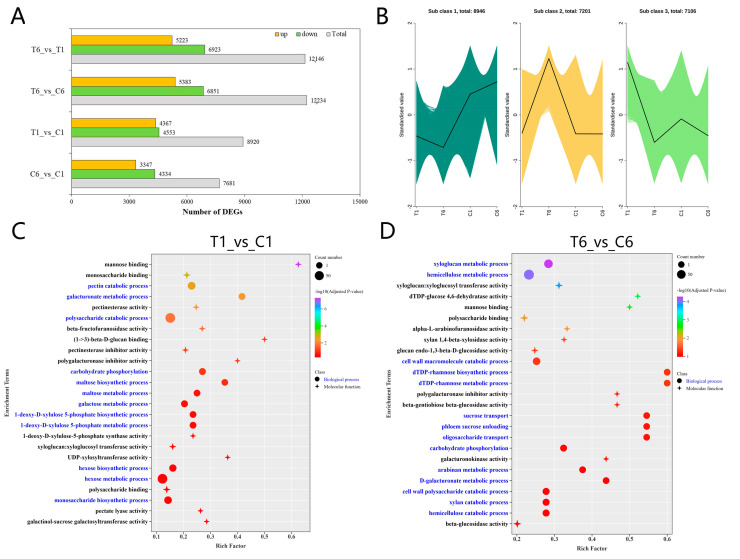
Clustering of all DEGs and GO terms related to polysaccharides in *P. cyrtonema* Hua. (**A**) The number of DEGs in the four compared groups. (**B**) The expression trends of DEGs by K-means clustering. (**C**) GO terms of T1_vs_C1. (**D**) GO terms of T6_vs_C6. The rich factor for genes is the transcriptome enrichment factor, which is the ratio of the number of differential genes annotated to the GO pathway to the number of background genes. The larger the rich factor, the greater the degree of enrichment. The larger the −log10 (Adjusted *p*-value), the more significant the enrichment.

To further clarify which genes may contribute to the high accumulation of polysaccharides in T6 rhizomes after plucking flowers, transcriptome analysis of T1, T6, C1, and C6 was performed. Raw reads were filtered to remove low-quality reads, and a total of 625,611,882 clean reads were generated. The Q30 values varied between 94.03~94.97%, and the GC contents were 48.09~49.16% (Table S2). The transcript N50 value was 1501 bp, and the gene N50 value was 1659 bp (Table S3). The total Benchmarking Universal Single-Copy Orthologs (BUSCO) score was 96.5% (Figure S1), indicating that these transcriptome data are trustworthy for further analysis. Data annotation showed that a total of 119,097 genes were obtained from the transcriptome sequencing, and 45.68, 61.07, 44.58, 60.61, 35.01, 52.31, and 38.67% of the genes were annotated as significant hits in KEGG, NR, Swissprot, TrEMBL, KOG, GO, and Pfam databases, respectively (Table S4). A two-group comparison method was adopted to identify differentially expressed genes (DEGs), and 23,253 DEGs were identified among the four groups. Specifically, 12,146, 12,234, 8920, and 7681 DEGs were identified in the T6_vs_T1, T6_vs_C6, T1_vs_C1, and C6_vs_C1 groups, respectively (Figure 4A). All DEGs were then categorized into three different expression patterns by K-means cluster analysis (Figure 4B). The gene expression pattern of subclass 2 (Figure 4B) showed a similar trend to that of total polysaccharide accumulation (Figure 1D) and metabolite profiles of subclass 5 (Figure 3A). To explore the possible DEGs involved in the regulation of polysaccharide biosynthesis in T6 rhizomes of *P. cyrtonema* Hua, polysaccharide-related GO terms were investigated further. GO term analysis showed that the molecular processes of “mannose binding” and “monosaccharide binding”, and the biological processes of “pectin catabolic process” and “galacturonate metabolic process” were enriched in the T1_vs_C1 (Figure 4C). In addition, the biological processes of “xyloglucan metabolic process”, “hemicellulose metabolic process” and “cell wall polysaccharide metabolic process”, and the molecular processes of “xyloglucan: xyloglucosyltransferase activity” and “dTDP-glucose 4,6-dehydratase activity” were enriched in T6_vs_C6 (Figure 4D). These results suggested that there exists a divergence in biological and molecular processes between T1_vs_C1 and T6_vs_C6 rhizomes.

#### 2.3.2. Gene Correlation Network Analysis and Transcription Factors

**Figure 5:**
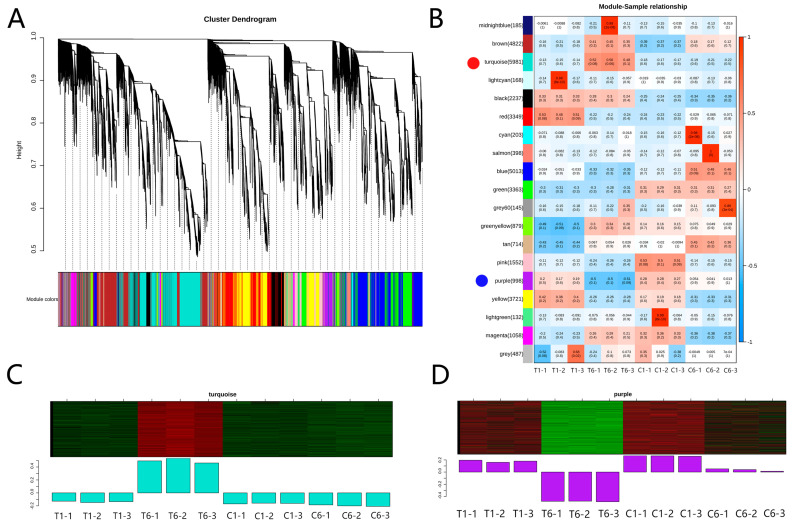
WGCNA analysis of DEGs among different groups. (**A**) Overview of the weighted correlation network analysis. (**B**) Relationship between modules and samples T1, T6, C1, and C6. (**C**,**D**) The genes from the turquoise and purple modules showed positive and negative expression patterns, respectively. In (**A**), each module color indicates one module, which includes the genes with similar expression patterns and the module threshold was set to 0.25, and the minimum number of module genes was 50. Red dots and blue dots in (**B**) represent the turquoise and purple modules, and the 19 major branches in the dendrogram (**A**) are included in (**B**). Heatmap from minus 1 to 1 indicates the correlation coefficient. Red is positively correlated and blue is negatively correlated. For example, 1 means that the correlation coefficient between the related genes is 100%. The two numerals in each module/block indicate the correlation coefficient and number of genes. In C and D, red denotes high expression, while green denotes low expression. The bar graphs indicate the expression values of the module eigenvalues for different samples.

Plant gene regulation is a complex process under stress or external environmental influence; thus, weighted gene co-expression network analysis (WGCNA) was performed using DEGs to identify gene co-expression networks for polysaccharide biosynthesis. Eventually, 19 major branches were obtained from the DEGs (Figure 5A,B). The turquoise and purple modules were positively and negatively associated with polysaccharide content in T6, respectively (Figure 5B). GO terms of the genes in the turquoise module were primarily associated with sugar transporters that may be involved in polysaccharide biosynthesis (Figure 5C, Table S5), while the genes in the purple module prominently included a few transferase genes that may be involved in polysaccharide biosynthesis/metabolism (Figure 5D, Table S6). Transcription factors (TFs) are key regulators that modulate the expression of target genes in plants. In the turquoise module, 163 genes were identified as TFs (Table S5). In the purple module, 92 genes were identified as TFs, of which the *XET* C-terminus (xyloglucan endo-trans glycosylase C-terminus, *cluster-33814.1*) belongs to the CCCH-type (C3H-type) TFs (Table S6). *XETs* play an important role in the depolymerization of plant structural polysaccharides, such as xyloglucans and cellulose, and are also involved in polymers and their cross-linking of newly generated components [14], which underlie plant cell wall dynamics and mechanics [15]. The C-terminus of *XET* provides an additional β-strand and a short α-helix, revealing a very favorable acceptor binding site [16]. It is possible that *XET* and the transferase genes in the purple module may negatively regulate cell wall remodeling jointly and contribute to the high accumulation of polysaccharides in T6 rhizomes.

### 2.4. Integrated Transcriptome-Metabolome Analyses

#### 2.4.1. Common KEGG Pathway Enrichment and Correlation Analysis of DEMs and DEGs

**Figure 6:**
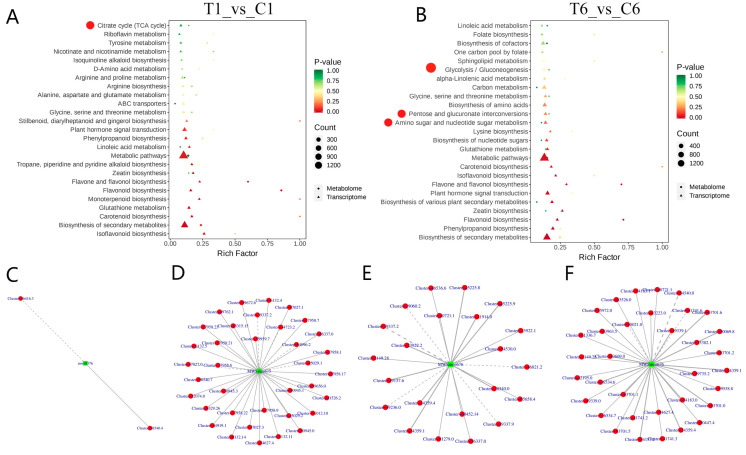
KEGG enrichment and correlation analyses of DEMs and DEGs. (**A**) KEGG enrichment of DEMs and DEGs from T1_vs_C1. (**B**) KEGG enrichment of DEMs and DEGs from T6_vs_C6. (**C**–**F**) Correlation network diagram displays the DEMs and DEGs from (**A**,**B**). Red dots in (**A**,**B**) represent the KEGG pathways related to polysaccharide biosynthesis; metabolites and genes in (**C**–**F**) are marked with green squares and red circles, respectively. The solid line represents a positive correlation, and the dotted line represents a negative correlation. mws0376 from the TCA cycle (**C**) represents fumaric acid; MWSmce675 from the glycolysis/gluconeogenesis pathway (**D**) represents arbutin; MWSmce676 from pentose and glucoronate interconversions (**E**) and amino sugar and nucleotide sugar metabolism (**F**) pathways represent d-arabinose.

To further determine the pathways involved in both DEMs and DEGs, common KEGG pathways were examined. The integrated transcriptome-metabolome analyses of T1_vs_C1 and T6_vs_C6 showed that, among the KEGG pathways related to the polysaccharide biosynthesis, the TCA cycle pathway was enriched in T1_vs_C1 (Figure 6A), whereas “glycolysis/gluconeogenesis”, “pentose and glucoronate interconversions” and “amino sugar and nucleotide sugar metabolism” pathways were enriched in T6_vs_C6 (Figure 6B). To further identify the key genes that lead to the changes in metabolites and that determine the key regulatory pathways, the correlation of DEMs and DEGs with Pearson correlation coefficient absolute value larger than 0.8 and *p*-value smaller than 0.05 in these four pathways (“TCA cycle”, “glycolysis/gluconeogenesis”, “pentose and glucoronate interconversions” and “amino sugar and nucleotide sugar metabolism”) were analyzed (Figure 6C–F). Results showed that in the TCA cycle, fumaric acid (mws0376) was associated with the phosphoenolpyruvate carboxykinase (*cluster-58540.4*, correlation = 0.946) and aconitate hydratase (*cluster-46416.3*, correlation = −0.806) (Figure 6C, Table S7). In the “glycolysis/gluconeogenesis” pathway, arbutin (MWSmce675) was significantly related to the phosphoglycerate kinase (*cluster-50945.3*, correlation = 0.931) (Figure 6D, Table S8). In the “pentose and glucoronate interconversions” and “amino sugar and nucleotide sugar metabolism” pathways, d-arabinose (MWSmce676) showed a strong positive correlation with pectinesterase (*cluster-54530.0*, correlation = 0.984) (Figure 6E, Table S9) and chitinase (*cluster-54183.0*, correlation = 0.994) (Figure 6F, Table S10), respectively. Among the above four pathways, the pathways “glycolysis/gluconeogenesis”, “pentose and glucoronate interconversions” and “amino sugar and nucleotide sugar metabolism” were particularly related to a polysaccharide unit (d-arabinose) under plucking flower treatment in T6_vs_C6, which was research-worthy. 

#### 2.4.2. DEMs and DEGs Related to Polysaccharide Synthesis 

**Figure 7:**
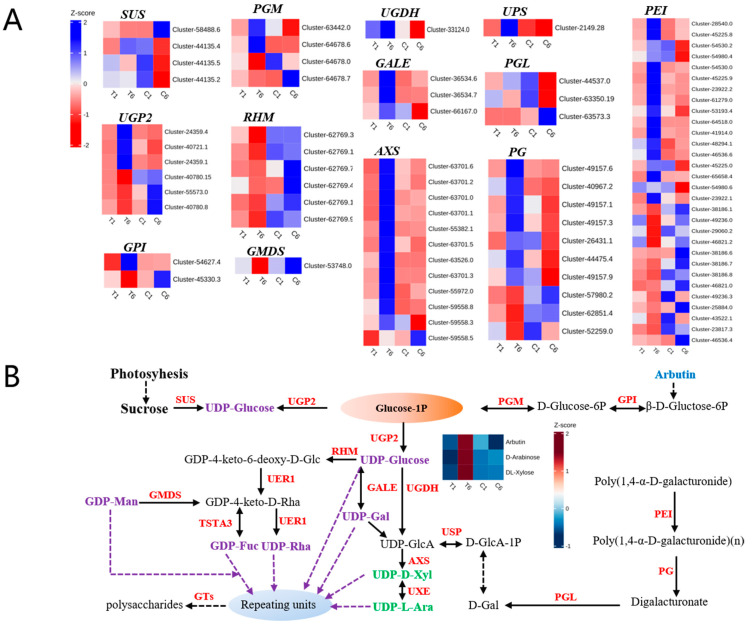
Heatmaps and pathways associated with polysaccharide synthesis in *P. cyrtonema* Hua. (**A**) Heatmaps illustrating major expression profiles of differentially expressed genes (DEGs) in T6_vs_C6. Z-scores in (**A**) are zero-means normalization of FPKM values. (**B**) Pathways and metabolism associated with polysaccharide synthesis. Gene names are in red, and the saccharides upregulated in T6_vs_C6 are in lime green, while other polysaccharide units are in purple. Arbutin is shown in blue for its function in glycolysis and gluconeogenesis. The heatmap in (**B**) indicates the contents of arbutin, d-arabinose, and dl-xylose in four samples. Z-scores are zero-means normalization of the peak areas of the metabolites. The solid arrows indicate a one-step reaction, and the dashed arrows represent multi-step reactions.

According to the previous and above studies [11,13], the “carbohydrate metabolism” subcategory, including “glycolysis/gluconeogenesis”, “pentose and glucoronate interconversions” and “amino sugar and nucleotide sugar metabolism” pathways was analyzed and genes related to polysaccharide biosynthesis in *P. cyrtonema* Hua were selected for further investigation (Figure 7A,B, Table 1). Sucrose synthase (*SUS*) catalyzes the reversible breakdown of sucrose to fructose and UDP-glucose [17]. Three *SUS* genes (*cluster-44135.4*, *cluster-44135.5,* and *cluster-44135.2*) were upregulated in C1_vs_C6, and one *SUS* (*cluster-58488.6*) was downregulated in T6_vs_C6. UTP-glucose-1-phosphate uridylyltransferase (*UGP2*) catalyzed the reversible conversion and regulated the balance between Glc-1-P and UDP-glucose [18]. Here, three *UGP2* genes (*cluster-40780.15*, *cluster-55573.0,* and *cluster-40780.8*) were upregulated in T6, and three *UPG2* (*cluster-24359.4*, *cluster-40721.1,* and *cluster-24359.1*) were downregulated in C6. Glucose-6-phosphate isomerase (*GPI*) is responsible for the reversible isomerization of glucose-6-phosphate (G6P) to fructose-6-phosphat (F6P) in glucose pathways, and *cluster-54627.4* and *cluster-45330.3* showed opposite expression patterns in T6_vs_C6 (Figure 7A). Phosphoglucomutase (*PGM*) catalyzes the interconversion of glucose 1-phosphate (G1P) and glucose 6-phosphate (G6P), which plays a vital role in linking glycolysis and gluconeogenesis pathways [19]. Two *PGM* genes (*cluster-63442.0* and *cluster-64678.6*) were upregulated, and *cluster-64678.7* was downregulated in T6_vs_C6. Moreover, UDP-glucose 4,6-dehydratase (*RHM*) and GDP-mannose 4,6-dehydratase (*GMDS*) were downregulated in T6_vs_C6. In contrast, UDP-glucose 6-dehydrogenase (*UGDH*), UDP-glucose 4-epimerase (*GALE*), UDP-apiose/xylose synthase (*AXS*), and UDP-sugar pyrophosphorylase (*USP*) were upregulated, which may contribute to polysaccharide accumulation (Figure 7A). Galacturan 1,4-α-galacturonidase (*PGL*), endo-polygalacturonase (*PG*), and pectinesterase (*PEI*) are involved in the cell wall pectin synthesis through the pentose and glucuronate interconversion pathways (Figure 7B). Heatmaps showed that the majority of these DEGs were upregulated in T6_vs_C6, and a minority of these DEGs showed opposite expression patterns in T6_vs_C6 (Figure 7A), which may be due to the large number of DEGs participating in the biosynthesis process of cell wall pectin. Further metabolite analysis revealed that two polysaccharide monomeric units (UDP-d-Xyl and UDP-l-Ara) accumulated in T6_vs_C6 (Figure 7B). These results suggested that both the DEGs and DEMs responded to plucking flower, and UDP-d-Xyl and UDP-l-Ara are important polysaccharide monomeric units in T6_vs_C6.

### 2.5. Validation of DEGs Related to Polysaccharide by qRT-PCR Analysis

**Figure 8:**
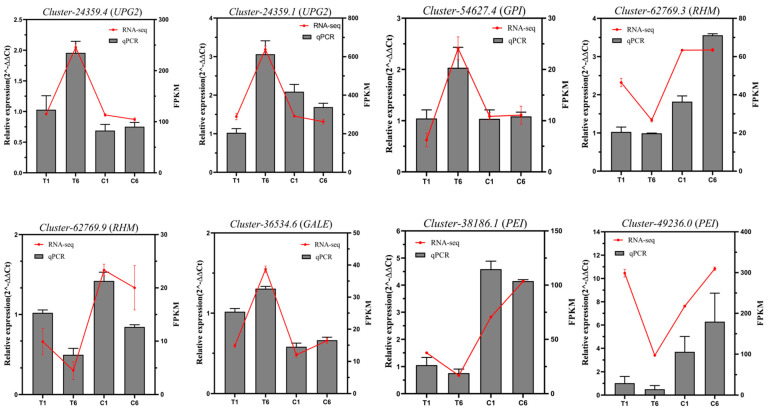
RNA-seq and qRT-PCR analysis of DEGs associated with polysaccharide synthesis in *P. cyrtonema* Hua. The gray histogram on the left y-axis represents qRT-PCR and the red line on the right y-axis represents FPKM.

Eight DEGs related to polysaccharide biosynthesis were subjected to validation of gene expression by qRT-PCR (Figure 7). The qRT-PCR results of these eight DEGs showed a similar expression pattern to the transcriptomic results, which validated the transcriptomic data (Figure 8).

## 3. Discussion

Polysaccharides have multiple functions [20] and are abundant in *Huangjing* [1]. According to the Chinese Pharmacopoeia (2020 edition), the polysaccharide content of the quantified PR was not less than 4%. However, the content of polysaccharides in rhizomes varied among germplasms, with the highest polysaccharide content in “*Baiji-type*” PR (BJPR) being 70.5 ± 5.37 mg/g, while the lowest in the “*Cylinder-type*” PR (CPR) was 48.5 ± 7.75 mg/g [21]. Another study showed that the polysaccharide content of rhizomes increased with age when seedlings were younger than four years old, and the polysaccharide content of four-year-old rhizomes of *P. cyrtonema* Hua was the highest [22]. In fact, the polysaccharide content of rhizomes of the majority of *P. cyrtonema* Hua was 70~100 mg/g, except that the polysaccharide content of rhizomes of *P. cyrtonema* Hua planted in Zhejiang Province was estimated to be higher than 150 mg/g, and the highest polysaccharide content was around 250 mg/g [23]. In this study, the polysaccharide content of the T6 rhizome increased significantly, reaching 201.1 ± 24.61 mg/g (Figure 1C), much higher than that of the C6 and C1 rhizomes. This phenomenon suggests that regardless of the effect of bacterial communities and soil properties on the active ingredients of *P. cyrtonema* Hua [23], it may be possible that agronomic practices can provide an alternative, convenient, and efficient way to dramatically increase the active ingredient level, especially the polysaccharide content. Our findings are consistent with previous studies showing that plucking flowers can increase the biomass and accumulation of effective substances in medicinal materials [8,9,24]. 

The accumulation of polysaccharide content in the T1 and T6 rhizomes treated with flower plucking was different, and it is necessary to clarify the role of the metabolites and genes involved. Therefore, metabolomics was performed, which provided some important clues. The total number of downregulated metabolites in T1_vs_C1 was greater than that in T6_vs_C6 (Figure 2C,D), suggesting that the 1Y rhizomes are more sensitive to the external environment due to their direct connection with the aboveground parts of the plant [10]. d-Fructose 6-phosphate and d-glucose 6-phosphate were downstream products of sucrose catabolism, and these two compounds decreased in T1_vs_C1. These results implied that 1Y rhizomes were directly influenced by plucking flowers (Figure 2E). However, the number of upregulated metabolites from T6_vs_C6 was greater than that of T1_vs_C1 (Figure 2C,D). This was probably because there was more alteration in DEGs related to the metabolites in the T6 rhizomes. Further results showed that flavonoids, phenolic acids, lipids, and others were the main metabolites identified (Figure 3B), and four saccharides including 4-*O*-galactopyranosylxylose, 6-*O*-α-l-arabinopyranosyl-d -glucopyranose, d-arabinose and dl-xylose were strongly associated with polysaccharide content in T6, which explains they could be the vital metabolites (Figure 3C). Overall, metabolomics provided visual data on polysaccharide accumulation between T1_vs_C1 and T6_vs_C6 rhizomes. 

Transcriptome analysis showed that the number of DEGs was similar to that of DEMs (Figure 2C and Figure 4A); therefore, some key genes may play critical roles in polysaccharide pathways (Figure 4B). GO term analysis showed that the “mannose binding”, “d -glucan binding”, “pectin catabolic process”, “galacturonate metabolic process”, “xyloglucan metabolic process”, “hemicellulose metabolic process”, “cell wall polysaccharide metabolic process”, “xyloglucan: xyloglucosyltransferase activity” and “dTDP-glucose 4,6-dehydratase activity” were the main processes affecting the polysaccharides, which agreed with the pathway classifications for carbohydrate metabolism [11]. WGCNA analysis revealed that the sugar transporters and the transferase family showed positive and negative correlations with polysaccharide content in T6 (Figure 5C,D, Tables S5 and S6). These results indicate that sugar transporters and transferases may play important roles in this process.

In the integrated transcriptome-metabolome analyses, we focused on the DEMs and DEGs of the “glycolysis/gluconeogenesis”, “pentose and glucoronate interconversions” and “amino sugar and nucleotide sugar metabolism” pathways (Figure 7A). In this study, plucking flowers significantly reduced the tensile force of flowers on carbon assimilation and strongly increased the tensile force of rhizomes on carbon assimilates, which was bound to promote the continuous flow of carbon assimilates to the rhizomes. Glucose-1P (Glc-1-P) is the critical substance involved in the three pathways simultaneously (Figure 7B). UDP-glucose pyrophosphorylase (*UGPase*) catalyzes Glc-1-P to UDP-glucose and plays important roles in controlling the strength of sink tissues [25] and in polysaccharide synthesis [18]. Previous studies have shown that the overexpression of *Larix gmelinii UGPase* enhances vegetative growth in transgenic *Arabidopsis thaliana* [26]. Thus, plucking flowers at the bud stage may have a similar effect in disrupting the balance between vegetative and reproductive growth and altering the direction of nutrient distribution. Here, we obtained six *UGPs* (Table 1), among which some were upregulated and the others were downregulated in T6_vs_C6 rhizomes (Figure 7A). This result may represent functional differences among *UPG* family members [27]. In addition, *UGDH*, *GALE,* and *USP* are also essential in modulating sugar metabolism. For example, *UGDH* is a key factor in the conversion of UDP-glucose to UDP-glucuronic acid [28], and overexpression of *LgUGDH* increases the content of soluble sugars and hemicelluloses and enhances vegetative growth and cold tolerance in transgenic *A. thaliana* [29]. *GALEs* are nucleotide sugar interconversion enzymes, and overexpression of the potato *GALE* genes *StUGE45* and *StUGE51* increased the galactose content in potato tuber cell walls [30]. Additionally, *USP* is essential for monosaccharide recycling and pollen development in *A. thaliana* [31]. The above results indicate that the upregulated *UGDH*, *GALE,* and *USP* genes are vital genes responsible for the increasing polysaccharide content such as d-arabinose and dl-xylose in T6 rhizomes of *P. cyrtonema* Hua, and are also responsible for vegetative growth after plucking a flower. 

In conclusion, plucking flowers contributed to a high accumulation of different types of polysaccharide units in T6 rhizomes of *P. cyrtonema* Hua. In addition, the polysaccharide content was much higher than the requirement for qualified PR, according to the Chinese Pharmacopoeia (2020 edition). The rhizomes of *P. cyrtonema* Hua are the most important harvested parts for both reproduction and human consumption [4]. The non-harvest parts like flowers (buds) are also of high nutritional value and good palatability and can be processed into beverages and food such as Huangjing flower teas. Overall, plucking flowers is a simple agricultural practice that is beneficial for the production of rhizomes and consumption exploration of flowers, thereby maximizing the profitability of the planting industry. 

## 4. Materials and Methods

### 4.1. Plant Materials

The experiment was conducted in the Huangjing planting base in 2023 in Qiping Town, Yichun City, Nanchang (28°67′49″ N, 114°19′64″ E), and Jiangxi Province, China. At the flower bud stage of *P. cyrtonema* Hua in April 2023, 60 plants were randomly selected based on the criteria of normal and uniform growth. Half of the plants were subjected to the removal of all buds, while the remaining plants served as a control group without any treatment. Each replicate contained 10 plants. One-year-old (1Y) and six-year-old (6Y) rhizomes from the control group were designated as C1 and C6, respectively. Similarly, one-year-old (1Y) and six-year-old rhizomes (6Y) of the plucking flower group were designated T1 and T6, respectively (Figure 1A). “1Y” represents one-year-old rhizomes of *P. cyrtonema* Hua, “6Y” represents two- to six-year-old rhizomes of *P. cyrtonema* Hua. Once the aboveground portion of *P. cyrtonema Hua* had wilted, the rhizomes were collected and frozen in liquid nitrogen, then stored at −80 °C for subsequent analysis.

### 4.2. Total Polysaccharides Content Analysis

Total polysaccharides were isolated from dried samples of C1, T1, C6, and T6 by the method of hot water extraction and ethanol precipitation [11]. In brief, 50 mg dried powder from each sample was mixed with 1 mL sterile water and extracted at 100 °C for 2 h. The samples were centrifuged at 10,000× *g* for 10 min to collect the supernatant. Then 0.2 mL of supernatant was mixed with 0.8 mL of absolute ethanol and left overnight at 4 °C. Finally, the samples were centrifuged at 10,000× *g* for 10 min to collect the precipitate, which was dissolved in 1 mL of distilled water. The supernatant (200 μL) was mixed with 100 μL Solution 1 supplied in the kit (Cat: M1505A, Michy Biology, Suzhou, China) and 500 μL sulfuric acid. The mixture was incubated in a 90 °C water bath for 20 min. Solution absorbance was then determined using a microplate reader at 490 nm. Glucose was used as the control, and the regression equation was as follows:

Y = 8.4038X + 0.0099(1)

Total polysaccharide content (μg/g dry weight) = (ΔA − 0.0099) × V1 × V3 × 1000)/(8.4038 × V2 × W)(2)

Equation (1): ΔA = Asample-Ablank. X is the glucose content (mg/mL), Y is absorbance A, R^2^ = 0.9968.
Equation (2): V1: re-dissolution volume, 1 mL; V2: alcohol precipitation volume, 0.2 mL; V3, water extraction volume, 1 mL; W: Sample quality, g; 1000, mg to μg conversion.

### 4.3. Sample Preparation and UPLC−MS/MS Metabolomics Analysis

The freeze-dried samples of C1, T1, C6, and T6 were ground (30 Hz, 1.5 min) to powder using a grinder (MM 400, Retsch, Düsseldorf, Germany). Then, 50 mg of powder was weighed and added to 1200 μL of −20 °C pre-cooled aqueous 70% methanol internal standard extract. Samples are vortexed six times for 30 s every 30 min. After centrifuging (12,000 rpm, 3 min), the supernatant was aspirated, each filtered through a microporous filter membrane (0.22 μm) and stored in the injection vial for UPLC−MS/MS analysis. 

The UPLC (ExionLC™ AD, https://sciex.com.cn/) and MS/MS (Tandem mass spectrometry) were used for UPLC−MS/MS analysis. The UPLC conditions were as follows: column, Agilent SB-C18 (1.8 µm, 2.1 × 100 mm); the mobile phase solvent A and solvent B were ultrapure water with 0.1% formic acid and acetonitrile with 0.1% formic acid, respectively. Sample measurements were performed with a gradient program that employed the starting conditions of 95% A and 5% B. Within 9 min, a linear gradient to 5% A and 95% B was programmed, and a composition of 5% A and 95% B was maintained for 1 min. Subsequently, a composition of 95% A, 5.0% B was adjusted within 1.1 min and kept for 2.9 min. The flow velocity was set as 0.35 mL per min, the column oven was set to 40°C, and the injection volume was 2 μL. The MS analysis conditions were as follows: source temperature 550°C; ion spray voltage (IS) +5500 V (positive ion mode) /−4500 V (negative ion mode); ion source gas I (GSI), gas II(GSII), and curtain gas (CUR) were set at 50, 60, and 25 psi, respectively; the collision-activated dissociation was high. QQQ scans were acquired as multiple reaction monitoring experiments with collision gas (nitrogen) set to the medium. DP (declustering potential) and CE (collision energy) for individual MRM transitions were performed with further DP and CE optimization. A specific set of MRM transitions was monitored for each period according to the metabolites eluted within this period.

For the two-group analysis, DEMs were determined by VIP (VIP>1) and absolute Log2FC (|Log2FC| ≥ 1.0). VIP values were extracted from the OPLS-DA results, which also contained score plots and permutation plots, and were generated using the R package MetaboAnalystR. The data were log-transformed (log2) and mean-centered before OPLS-DA. In order to avoid overfitting, a permutation test (200 permutations) was performed. Metabolomic analysis was performed using Metware Cloud (https://cloud.metware.cn, accessed on 1 July 2024).

### 4.4. RNA-seq Analysis

Total RNA was extracted by ethanol precipitation and CTAB-pBIOZOL and quantified using a Qubit fluorescence quantifier (Qubit 4.0, Thermo Fisher Scientific, Waltham, MA, USA) and a Qsep400 high-throughput biofragment analyzer (Qsep400, Bioptic, Shanghai, China). cDNA libraries were sequenced on an Illumina sequencing platform by Metware Biotechnology Co., Ltd., (Wuhan, China). Low-quality (Q < 20) reads were removed to obtain clean reads. Transcriptome assembly of clean reads was performed using Trinity (v2.13.2), the assembled transcripts were clustered, and redundant reads were removed using Corset (1.09) (https://github.com/trinityrnaseq/trinityrnaseq, accessed on 1 July 2024). The longest cluster obtained by Corset hierarchical clustering was determined to be the gene for further analysis.

The expression level of transcripts was calculated using RSEM software (v1.3.1), and then the fragments per kilobase of transcript per million mapped reads (FPKM) of each transcript was calculated according to the transcript length. Differential expression analysis between the two groups was performed using DESeq2 [32]. The criteria for identifying DEGs were |log2Fold Change| ≥ 1 and FDR < 0.05. A *P*-value threshold of ≤ 0.05 was used to identify GO entries with significantly enriched DEGs for functional annotation [33]. The function analysis (GO) was performed using the Metware Cloud (https://cloud.metware.cn, accessed on 1 July 2024).

### 4.5. WGCNA

Transcriptome data for 12 samples were normalized to FPKM to select genes for gene co-expression network analysis. Key modules that might be related to the polysaccharide content in *P. cyrtonema* Hua were identified according to the correlations between modules and traits.

### 4.6. Integrated Analyses of Transcriptomic and Metabolomic Data

To screen and obtain the metabolites and genes that influence samples, common pathways shared by metabolites and genes in two groups (T1_ vs_C1 and T6_ vs_C6) were described in a bubble chart, and the correlation network was visualized using Cytoscape (version 2.8.2).

### 4.7. Quantitative Real-Time Reverse Transcription PCR (qRT-PCR) Analysis

Total RNA was treated with DNase I to remove genomic DNA and then reverse-transcribed to obtain first-strand cDNA using the RevertAid RT enzyme (Thermo Scientific) according to the manufacturer’s instructions. To verify the RNA-seq data, candidate genes were determined using qRT-PCR with the PerfectStart Green qPCR SuperMix (AQ601-02-V2, TransGen, Beijing, China). The *UBQ-E2-10* gene was used as a reference to normalize the expression of target genes [34,35]. All primer pairs were designed using Primer Premier 5 (Table S11). All genes were repeated in three biological and technical replicates. The expression level was calculated by the 2^−ΔΔCt^ method [36].

### 4.8. Statistical Analysis

Three replicates were performed using GraphPad Prism 8.0, and data are shown as mean ± SEM. Significance was assessed by the *t*-test. *p*-value < 0.05 was considered to be statistically significant (* *p* < 0.05; ** *p* < 0.01 and *** *p* < 0.001, **** *p* < 0.0001.).

## Figures and Tables

**Table 1:** The genes related to polysaccharide biosynthesis.

Gene Symbol	Gene Name	EC	Number of Genes
*SUS*	Sucrose synthase	EC:2.4.1.13	4
*UGP2*	UTP-glucose-1-phosphate uridylyltransferase	EC:2.7.7.9	6
*GPI*	Glucose-6-phosphate isomerase	EC:5.3.1.9	2
*PGM*	Phosphoglucomutase	EC:5.4.2.2	4
*RHM*	UDP-glucose 4,6-dehydratase	EC:4.2.1.76	6
*GMDS*	GDP-mannose 4,6-dehydratase	EC:4.2.1.47	1
*GALE*	UDP-glucose 4-epimerase	EC:5.1.3.2	3
*UGDH*	UDP-glucose 6-dehydrogenase	EC:1.1.1.22	1
*AXS*	UDP-apiose/xylose synthase	EC:4.1.1.35	12
*USP*	UDP-sugar pyrophosphorylase	EC:2.7.7.64	1
*PGL*	Galacturan 1,4-α-galacturonidase	EC:3.2.1.67	3
*PG*	Endo-polygalacturonase	EC 3.1.1.15	10
*PEI*	Pectinesterase	EC:3.2.1.11	30

## Data Availability

Data are available within the article and Supplementary Materials.

## Supplementary Materials

The following supporting information can be downloaded at: www.mdpi.com/article/10.3390/molecules30030670/s1. Figure S1: BUSCO assessment of *P. cyrtonema* Hua; Table S1: 657 DEMs in all four compared groups; Table S2: Sequencing data quality of *P. cyrtonema* Hua; Table S3: Assembly of *P. cyrtonema* Hua genes; Table S4: Summary of *P. cyrtonema* Hua genes annotated in seven public databases; Table S5: in the turquoise module; Table S6: Genes in the purple module; Table S7: Genes and meta correlation in TCA cycle; Table S8: Genes and meta correlation in glycolysis/gluconeogenesis pathway; Table S9: Genes and meta correlation in pentose and glucuronate interconversions pathway; Table S10: Genes and meta correlation in amino sugar and nucleotide sugar metabolism pathway; Table S11: Primers used for qRT-PCR.
