# Supplementary figures and images for: Integrative Metabolome and Transcriptome Analyses Reveal the Effects of Plucking Flower on Polysaccharide Accumulation in the Rhizomes of Polygonatum cyrtonema Hua
Source: Molecules. 2025 Feb 3;30(3):670. doi: 10.3390/molecules30030670 (PMC11820947; doi:10.3390/molecules30030670)

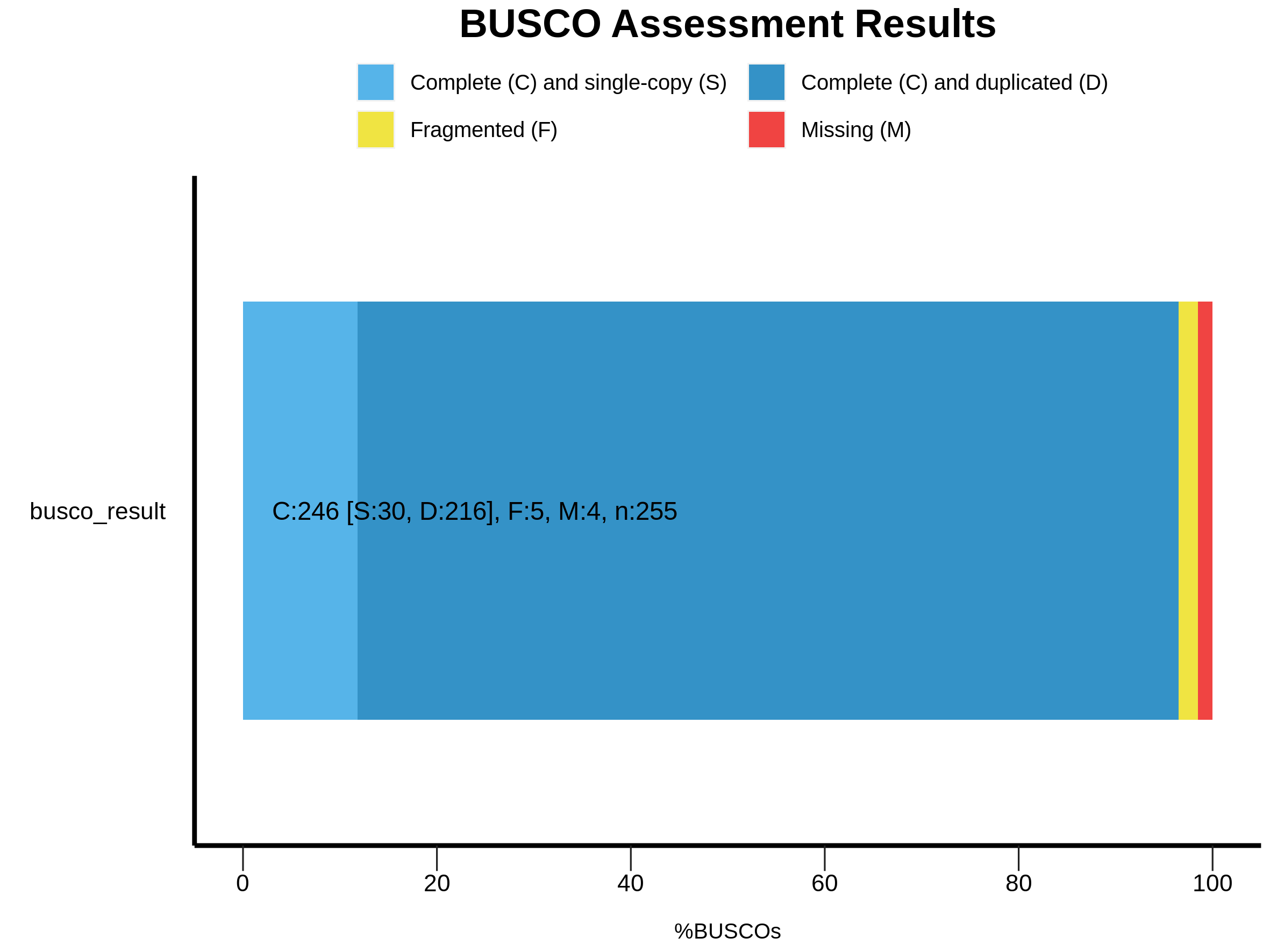

Supplement: Supplementary file 1 [file molecules-30-00670-s001.zip › Figure S1.png]
